# Parent and facilitator experiences of an intensive parent and infant programme delivered in routine community settings

**DOI:** 10.1017/S146342361900029X

**Published:** 2019-08-19

**Authors:** Yvonne Leckey, Gráinne Hickey, Ann Stokes, Sinéad McGilloway

**Affiliations:** 1 Currently (and at time of research) Researcher with ENRICH Research Programme, Maynooth University Department of Psychology, Maynooth University, County Kildare, Ireland; 2 Currently (and at time of research) Research Programme Manager with ENRICH Research Programme, Maynooth University Department of Psychology, Maynooth University, Maynooth, County Kildare, Ireland; 3 Currently (and at time of research) Postdoctoral Researcher with ENRICH Research Programme, Maynooth University Department of Psychology, Maynooth University, Maynooth, County Kildare, Ireland; 4 Currently (and at time of research) Director of the Centre for Mental Health and Community Research at Maynooth University, Maynooth University Department of Psychology, Maynooth University, Maynooth, County Kildare, Ireland

**Keywords:** community-based practice, early intervention, infant socioemotional development, parent and baby programme, parent–child relationship, public health nursing

## Abstract

**Aim:**

The aims of this study were to (1) assess the initial experiences of parenthood amongst mainly disadvantaged mothers; (2) explore their views on the extent to which they felt they had benefitted (or not) from participating in a newly developed, intensive mother and baby support programme in the community; and (3) explore the perspectives of those who delivered the programme (i.e., facilitators), most of whom were Public Health Nurses (PHNs).

**Background:**

Positive parent–child interactions and appropriate levels of infant stimulation are essential to promoting a child’s well-being and laying a foundation in the early years for positive developmental outcomes. It is important, therefore, to examine participants’ experiences of community-based, family-focused, early prevention and intervention programmes.

**Methods:**

This study was undertaken as part of a larger evaluation of a newly developed parent and infant (PIN) programme which was delivered in two disadvantaged areas in Ireland. One-to-one interviews were conducted with both mothers (*n* = 22) and facilitators (*n* = 8) (including three PHNs) plus six focus groups with an additional sub-group of facilitators (*n* = 17).

**Findings:**

The collective findings suggest that mothers found the programme helpful in promoting a greater understanding of their infants’ behaviour and needs, and in alleviating stress and concerns associated with motherhood. Mothers described feeling more knowledgeable about the importance of regular and appropriate infant interaction to encourage learning and development. Facilitators, specifically PHNs, also reported a greater awareness of the value of infant socioemotional development for their clinical practice and observed greater positive communication between mothers and infants.

**Conclusion:**

These findings suggest that a community-based, intensive mother and baby programme can help to promote parental competence and enhance infant learning and development. Additional benefits in terms of early intervention and positive changes to public health nursing practice are also discussed.

## Introduction

The earliest stages of parenthood can be challenging with important implications for the parent–child relationship and child development (Deave, Johnson & Ingram, 2008). While motherhood may represent a time of great joy and fulfilment, it can also be a stressful and anxious time as mothers adjust to changes in lifestyle and routines (Krieg, 2007). Mental health difficulties such as postnatal depression and anxiety can also interfere with a mothers’ ability to sensitively care for her infant (Haga *et al*., 2012; Sockol& Battle, 2015) and may give rise to higher levels harsh or disengaged parenting (Turney, 2011). An infant’s early development depends, in large part, upon a positive and affectionate parent–infant relationship that is sensitive and responsive to a child’s needs and lays the foundation for the child’s physical, cognitive and language development (Walker *et al*., 2011). Evidence has shown that brain development and the parent–infant relationship are inherently intertwined; growth of the infant brain supports the development of the parent–infant relationship, while the parent and surrounding environment, mould infant neurodevelopment (Malekpour, 2007; Parsons *et al*., 2010). These shared early experiences can provide lifelong health benefits and are fundamental to the development of social and emotional well-being in children (Bagdi & Vacca, 2005; Shonkoff & Richmond, 2009). A greater understanding of how adverse and positive experiences affect brain development and subsequent health and well-being outcomes has led to an increasing focus on early intervention and prevention programmes to support the mental health of children and mothers during the earliest (postnatal) years (Barlow *et al*., 2010a).

Universal access to high-quality services, particularly within areas of higher socioeconomic deprivation, is recommended in order to reach the most vulnerable children and promote health equity (Marmot *et al*., 2012; Black *et al*., 2017). Many countries have developed, or are in the process of developing, policies and initiatives to tackle developmental inequality and disadvantage which are aimed at promoting mental health and well-being in families (e.g., Early Head Start, Sure Start, and the Healthy Child Programme). In Ireland, recent policy initiatives (e.g., *Better Outcomes, Brighter Futures. The national policy framework for children and young people 2014–2020*, Department for Children and Youth Affairs, 2014) have focused on the need for evidence-informed early intervention and prevention services in order to achieve better long-term social and economic outcomes. Early years parenting strategies that are delivered on an area-wide basis and promote child outcomes and nurturing parenting skills, particularly for those most at risk, are the cornerstone of these policies. A significant body of evidence highlights the importance of positive parenting and parent–infant relationships for the future well-being of the child, whilst group-based parenting programmes for older infants and toddlers have been found to improve the socioemotional and behavioural adjustment of children (Olds, Sadler and Kitzman, 2007; Shonkoff & Richmond, 2009; Barlow *et al*., 2010b; Moullin, Waldfogel & Washbrook, 2014). However, gaps remain in our knowledge of the universal effectiveness of these programmes for parents and their infants from the earliest life stages (Pidano & Allen, 2015). Furthermore, there remain significant challenges to ensuring that families engage with, and receive, appropriate, effective and timely supports, particularly during the critical first 1000 days of a child’s life (Winston and Chicot, 2016).

This study was undertaken as part of a larger evaluation of a newly developed parent and infant (PIN) programme which is designed to build parenting competency, promote secure parent–child attachments and promote infant socioemotional development. The programme is inspired by wraparound principles (Bruns, Suter & Leverentz-Brady, 2008) and comprises a range of group-based supports, delivered to parents over the first two years of their child’s life, in collaboration with community-based services. The larger evaluation of the programme, which is still ongoing, involves a non-randomised controlled trial/impact evaluation, a process evaluation and a cost-effectiveness analysis (ENRICH (EvaluatioN of wRaparound in Ireland for CHildren and families)). The aims of the sub-study reported here, which was conducted as part of the embedded process evaluation, were to (1) to examine mothers’ experiences of early parenthood; (2) investigate their experiences and views of the PIN programme including their reasons for taking part, any perceived benefits and any difficulties/challenges; and (3) explore the experiences and views of those who were delivering the programme (i.e., facilitators).

## Method

### Participants and settings

Purposive sampling was used to recruit key stakeholders involved in the programme, including programme facilitators and parent participants. All parent participants were mothers who were identified and recruited by PHNs at local health centres, or through Family Resource Centres (i.e., who had identified new mothers in need of parenting support). No fathers had participated in the impact evaluation. A sub-sample of 26 mothers (across both sites) who had previously consented to participate in the impact evaluation (n = 190) were contacted by telephone and provided with information. To obtain a wide range of views, a mix of agesand first-time and second-time mothers were included and 22 of the 26 mothers consented to an interview. Of those who declined to participate, two mothers stated they were too busy and the remaining two were not interested. At the time of interview, all mothers, with the exception of one, were attending the programme. All interviews were conducted during a two-year period (2015–2017).

A total of 25 facilitators from both sites were interviewed, 17 of whom were PHNs and who were interviewed as part of six focus groups due to practical considerations related to early implementation and competing work schedules/commitments. The remaining eight facilitators were interviewed on a one-to-one basis; three were PHNs and the remaining five were Development Officers or Support Workers from community organisations tasked with programme delivery. Interviews with parent participants were conducted in the home. Facilitator interviews and focus groups were held at local health/community centres.

## The intervention

**Figure 1.**
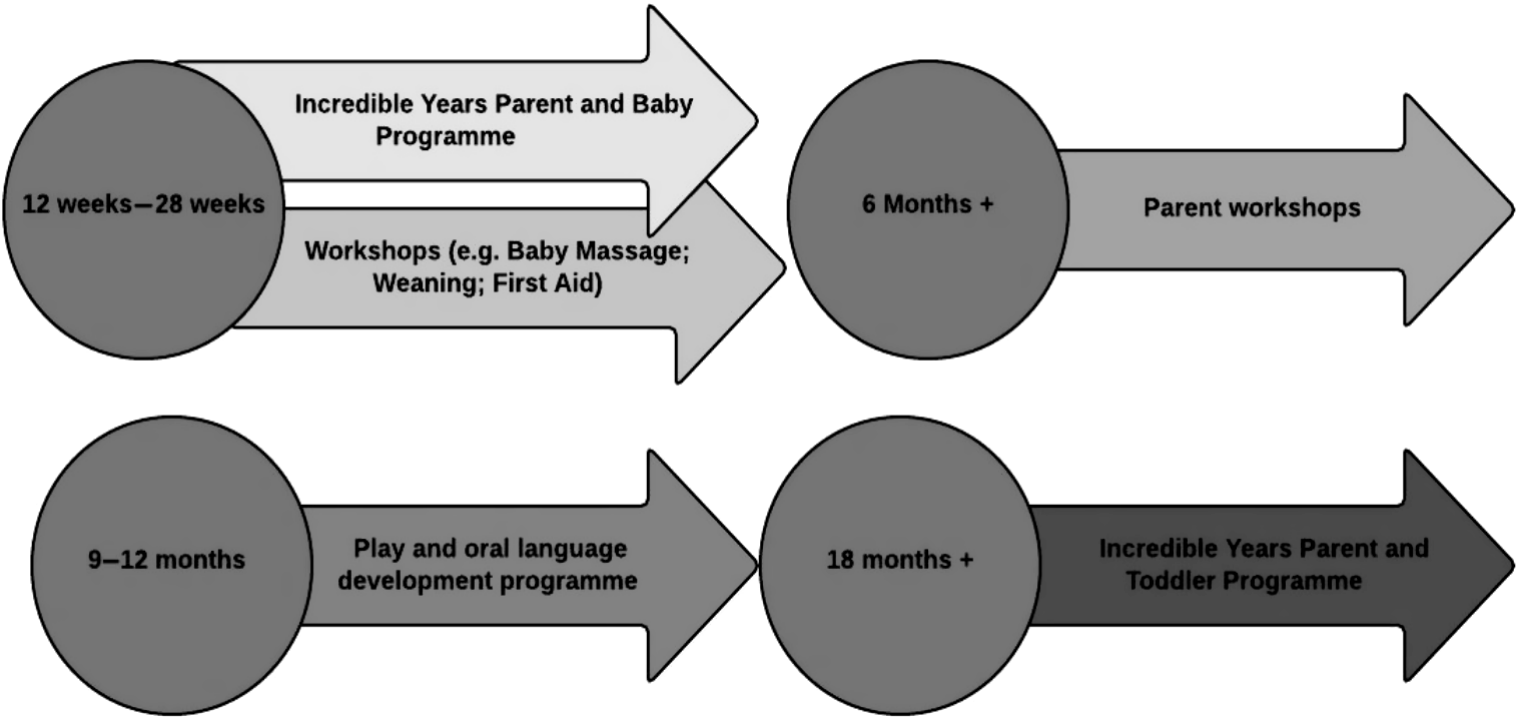
Parent and infant (PIN) programme components (according to age)

The PIN programme was developed by a non-profit community organisation, called Archways, in collaboration with Public Health Nurses (PHNs) and other community-based organisations in two sites in Ireland; both of which are located in urban areas in the east of the country. The programme comprises an intensive community-based intervention consisting of the Incredible Years (IY) Infant (IYBP) and Toddler (IYTP) Programmes (Webster-Stratton & Reid, 2011) plus a number of PIN supports that aim to promote parents’ sense of competence and well-being and encourage positive infant health and development. The first phase of the intervention is offered to parents when infants are between 2 and 6 months old and comprises of the IYBP alongside the baby massage, weaning and First Aid workshops. The second phase is offered to parents once the child is approximately 18–28 months old where a play/oral language development workshop, a healthy eating workshop and/or the Incredible Years Parent and Toddler Programme (IYPTP) is delivered. The various supports/workshops are all delivered in line with baby’s development (Figure 1).

Typically, mothers and babies commence the programme when the baby is aged 6–20 weeks old. Both IY programmes are rooted in behavioural and social learning principles and use DVD-modelling, group discussions and role play to promote positive attachment, as well as strategies to enhance baby’s physical, socioemotional and language development (Webster-Stratton & Reid, 2011). Babies attend all programme/workshop sessions with their mothers with the exception of the IYTP, weaning/healthy eating and first aid workshops. Research on the IYBP is limited to date, with some mixed findings; for example, short-term improvements have been found for parental mental health and confidence (Evans *et al*., 2015) and sensitive parenting (Jones, Erjavec, Viktor & Hutchings, 2016) while a more recent study (Pontoppidan, Klest & Sandoy, 2016) found no intervention effects. Hutchings *et al*., reported significant improvements for the IYTP in terms of parental well-being and parental praise at 6 months with further improvements demonstrated for parental well-being, child conduct and home environment at 12 months follow-up (Hutchings, Griffith, Bywater & Williams, 2017). It is hoped that a combination of the IY programmes and additional wraparound sessions will optimise health and well-being outcomes for both parents and infants.

## Measures

A number of semi-structured interview schedules and topic guides were devised for use in data collection, and interviews plus focus groups were conducted by four (female) researchers who were also involved in data collection for the larger evaluation. All researchers had a background in psychology or other social science discipline and had extensive experience conducting qualitative research in the area of parenting supports. For parent participants, an interview schedule was designed to capture their transition to motherhood and experiences of the programme including early mothering experiences, perceptions of changes in self, impact on home life, lifestyle and relationships. Parents were also asked what they found most useful/less useful about the programme and whether they had changed, or adopted, new practices following their participation. Interviews took 25–45 minutes to complete and parent participants were given a €15 shopping voucher in return for their participation. Similar to the parent interviews, an interview schedule and topic guide for facilitators were designed, respectively, to elicit their experiences of delivering the programme and perceived benefits or challenges both for them and for the mothers. The duration of one-to-one interviews ranged from 30 to 60 minutes. Focus groups (duration 30–40 minutes) were also conducted with facilitators across both sites. All parent interviews were conducted in the home settings whereas facilitator interviews were undertaken in health centres and clinics. All participants provided their written informed consent to take part and for the sessions to be recorded.

## Data analysis

Interviews were audio-recorded with consent, password protected and stored electronically on a secure server. All interviews were transcribed fully, and the data were transferred to MAXQDA. Transcripts were read repeatedly and initially analysed by first author using thematic analysis (Braun & Clarke, 2006). Thereafter, a line-by-line inspection of all data was undertaken to generate dominant themes, and a number of sub-themes were subsequently identified, and cross-checked, across both parent and facilitator groups. To increase reliability, regular meetings were held amongst research staff where themes were discussed, and interpretations of the data were compared and consensually validated. The study was guided, in part, by the Consolidated Criteria for Reporting Qualitative Studies (COREQ) (Tong, Sainsbury & Craig, 2007).

## Results

### Participant characteristics

A mix of primiparous and multiparous women (17 first-time and 5 second-time mothers, respectively) agreed to take part in the programme and in the research, and ranged in age from 18 to 41 years (Mn = 30; SD = 6.7) prior to programme commencement (baseline). Infants ages ranged from 3 to 30 months (Mn = 16.9) at time of interview. Prior to becoming pregnant, over half (*n* = 12) were working full-time, whilst the remainder were equally divided according to part-time working status or not working. Most participants were married/cohabiting (*n* = 17), while five were lone parents. Over half of the participants (*n* = 12) held a degree or professional qualification. Fourteen participants (74%) were considered disadvantaged (Table 1), based on a score of two or more of the following variables; lone parent, unemployment, large family (>3 children), early school leaver, anti-social environment and criminal activity.

**Table 1.** Demographic parent participant characteristics

	*N* = 22	%
Age		
18–22	3	14%
23–29	4	18%
30–35	8	36%
36–40	6	27%
41+	1	5%
Marital status		
Married/co-habiting	17	77%
Single	5	23%
Maternal education		
Degree/professional qualification	12	55%
Completed school	9	41%
Early school leaver	1	5%
Maternal work status (prior to becoming pregnant)		
Full-time	12	54.5%
Part-time	5	22.7%
Unemployed	5	22.7%
Socioeconomic disadvantage score*		
≥2/6	14	74%

* Socioeconomic disadvantage was based on a score of two or more of the following variables; lone parent, unemployment, large family (>3 children), early school leaver, anti-social environment, criminal activity.

Twenty-five facilitators were interviewed as part of the study. Five facilitators were Development Officers or Support Workers from community organisations with extensive experience in providing child and family support and facilitating IY programmes. The remaining facilitators were PHNs. Of the facilitators who participated in one-to-one interviews (*n* = 8); six were accredited IYBP group leaders and two were accredited IY peer coaches. Most participating facilitators were PHNs and worked in community-based primary care settings.

### Programme attendance and reasons for non-participation

The majority of interviews were conducted while participants were attending the IYBP, hence the analysis presented here focuses primarily on participants’ experiences during the early stages of the PIN programme. Some mothers (*n* = 2) interviewed had attended the Toddler (IYTP) programme which commenced when infant reached 24–26 months, the remaining mothers were either due to attend or unable to owing to work commitments and/or a lack of childcare. In order to contextualise participants’ experiences, the following attendance figures were analysed post-programme and give an indication of participation for the IYBP; 19 attended 5 or more sessions (out of a total of eight sessions) with two participants attending four sessions or less; 15 participants attended four of the seven complementary workshops or more, with 3 attending three sessions or less. One parent did not attend the programme citing a lack of transport/venue inaccessibility as the reason.

### Parents’ experiences

Three major themes (and several sub-themes therein) were identified from the analysis of parent participants (Table 2).

**Table 2.** Main themes and sub-themes related to parent participants

**Challenges associated with transitioning to motherhood**
Feeling unprepared, anxious and fearful
Mental health and well-being
Social isolation
**Experiences of programme participation**
Social / peer support
Gaining knowledge and new skills and importance of peer-led learning
Heightened awareness of infant development
**Programme challenges**

**1. Challenges associated with transitioning to motherhood**

***(a) Feeling unprepared, anxious and fearful***

The first few weeks post-birth can be a difficult time for mothers as they adjust to caring for a new born and themselves, as well as managing their relationships with their partner and family. Some mothers described their groundwork and planning for this role, including attending antenatal classes and gathering information on the practicalities of caring for their baby, in ensuring they were as fully prepared for motherhood as possible. Despite this, adapting to their new circumstances proved to be more difficult than anticipated, with many expressing a great deal of fear and self-doubt in caring for their baby:

*At the beginning, I found it very tough. I was kind of like why hasn’t anyone told me how tough this it? Nobody told me how hard it was going to be*. (P8)

*I thought it was an overwhelming change. I thought it totally changed our whole life in a positive way but there isn’t any book can prepare you for it*. (P4)

As expected, the effects of sleep deprivation, fatigue and the ‘on-demand’ infant feeding routines were widely reported as being challenging. Some mothers were still uncomfortable and exhausted after labour leaving them feeling increasingly overwhelmed and anxious:

*So I wish somebody had told me that at the start that it will get easier … I think it’s just a lot to deal with. You’re so sore after the labour, you’re absolutely exhausted, you’re very vulnerable and you’re not getting any sleep and everything as you know it has changed*. (P16)

*Sleep deprivation because I was on my own … that was the hardest part. To function for the day with sleep deprivation and with no support*. (P21)

***(b) Mental health and well-being***

Some mothers cited psychological issues, such as depression and stress, which they felt affected their sense of confidence and their ability to adequately care for their baby:

*When I was here on my own with him … I used to feel that I was driving myself nuts because I was talking to a child that wasn’t talking back to me*. (P14)

Loss of identity was also frequently mentioned by some mothers as they tried to balance their infant’s needs with that of being a new mother. These changes were particularly notable for mothers who were working prior to baby’s birth. Over three-quarters of the sample worked full, or part-time before baby’s arrival, and transitioning from the workplace to the home environment proved to be a significant challenge for some mothers:

*I don’t know if you’re ever prepared for it. It’s a huge change. Going from kind of your career … it’s about you and your partner and it was just the two of us and you didn’t really have to think about someone and now this person then becomes your entire life*. (P6)

***(c) Social isolation***

A recurring theme amongst participants related to the sense of social isolation felt by many mothers during the early postnatal months. Data from our impact evaluation showed that over a third of mothers within the larger sample (n = 106) reported feeling lonely since baby’s arrival. The qualitative findings reported here support and amplify this finding by highlighting how mothers felt housebound and overwhelmed with the practicalities of caring for their infant, and having limited time to themselves or with others:

*In the beginning I found it hard and a little bit overwhelming to do anything other than feed and look after baby*. (P16)

The majority of mothers in this sample spoke of their partners, family members, and particularly their own mothers, as an important source of support; however a sub-sample (*n* = 5) of mothers reported a lack of support from their wider family circle/friends which compounded their experiences in the earliest stages of motherhood:

*It’s very stressful and I didn’t find it all too easy. My first time … and I didn’t really have all the support here …* (P18)

**2. Experiences of programme participation**

Seven mothers reported attending the programme on a recommendation from their PHN. Participating mothers reported positive experiences of the programme and felt that they had benefitted from their participation in several ways: through social/peer support; gaining knowledge and new skills; the importance of peer-led learning; and a heightened awareness of the importance of promoting infant socioemotional development. Mothers found the programme helpful for getting to know their baby and for recognising the needs of infants and encouraging learning and development. They were very positive about the programme materials and workshops, with baby massage, weaning/toddler healthy eating and first aid proving to be popular amongst participants. Many parents reported benefits associated with baby massage both for bonding with baby and facilitating sleep:

*I felt very connected with him when we were doing it*. (P2)

*The massage was very, very good. Each time I did that, he slept for a longer period*. (P18)

Furthermore, second-time mothers also reported gaining new knowledge and skills around infant health and development. For example, one parent reported how the programme provided her with additional information on how to respond promptly and appropriately to her infant’s cues:

*… I am aware more about stuff. Because before it was like why are they crying? I will just try everything. And then I got the facial cues and the tongue and stuff like that so I was able to do things like feed her before she’d start crying*. (P1)

Another second-time mother described the changes to nutritional practices in the home as a consequence of attending the weaning workshop:

*But when I went and done the healthy eating food course, she just basically took out a potato, carrot, and something else like and she made it up. We tasted it and she made us taste that and the jar of baby food and I was like ‘oh my God’ and he’s never had baby food since*. (P10)

Overall it was felt that the various programme resources, in addition to the group support, enhanced parenting knowledge and engendered feelings of competence and confidence:

*I think it’s so important to have that support for mothers and it did shock me in the hospital that when you’re discharged you get a quick wave and a bye-bye and it’s all up to you now. And aside from those checks with your doctor, there’s literally nothing else out there. So I think a programme like this that helps you get out and meet other mothers or ask questions and share experiences and then actually learn some things along the way, it’s hugely valuable*. (P16)

*All of it was pretty good. They had a lot of things covered with the baby massage and the weaning that interlinked with it, and there was a lot of information in the actual group and there was video recordings and stuff like that so they kind of covered everything*. (P1)

***(a) The perceived benefits of social/peer support***

For the majority of participants, the importance of connecting with other mothers was a key incentive for programme participation. The groups provided a source of informal knowledge and support where mothers could openly express their frustration or anxiety and exchange advice on parenting needs. All mothers who attended the programme spoke of the benefits of sharing with other mothers, both for normalising their experiences and enabling them to regain control of their lives. These new connections and interactions helped to promote confidence in their parenting techniques:

*Meeting new mums and getting information that you don’t know as a first-time mother … because you think you’re the only one so it’s good to be able to be able to talk to people*. (P13)

The support and feedback from other parents were also crucial in promoting engagement. This was important in terms of alleviating social isolation by providing emotional support during the more difficult early postnatal months, as well as increasing the likelihood of facilitating continued engagement with the group over the longer term:

*I loved meeting the other mammies. I loved the interaction*. (P12)

Notably, social media was instrumental in providing constant reassurance and comfort for participants. All mothers reported regular use of apps, such as Facebook and WhatsApp, to maintain contact with other group members and to seek advice and support during stressful or difficult periods (e.g., sleepless nights, feeding difficulties or returning to work):

*You just feel like you’re more plugged into a network, plugged into a community and on a very practical level you can chat to other mums and say does your baby do this? … You feel less alone and isolated*. (P16)

***(b) Building knowledge and new skills and the importance of peer-led learning***

A desire to acquire new parenting knowledge and skills was identified as another reason for programme participation. Some mothers indicated that the programme provided them with useful information on common parenting issues and increased their confidence in their abilities to predict their infant’s needs and encourage their development. Notably, first-time mothers, with little practical experience in caring for babies, felt that they benefitted hugely from the programme:

*I didn’t know where to start with [baby] and doing the programme helped me a lot like. Helped me in different ways like. I was always the person with the question!* (P7)

Many mothers reporting feeling more capable as a parent with one mother pointing out: *‘I feel like I am a better mother since the programme’* (P5). It appeared that the mix of both first first-time mothers and second-time mothers worked well, with one participant valuing the group as a good source of information as *‘it was good to mingle with other mothers to find out all their tips’* (P17). Specifically, group discussions provided a valuable opportunity to listen to others’ views, problem solve and learn about new and/or different parenting techniques:

*So that was kind of nice to hear of someone who’s gone through it before. But even the other first-time mums, everyone develops at a different rate, so you know somebody might have done something or somebody would come in with a question and we’d kind of brainstorm it out*. (P6)

While the initial months were widely acknowledged as more stressful and demanding than expected, many mothers felt that as the programme progressed, and they became more familiar with their infants’ needs and became more confident and competent parents:

*When it’s a new-born it is an important relationship, you’ve got to meet their every need, you’ve got to anticipate what they want … but now it’s much more of a two-way thing. I know what she enjoys now … I suppose now I can predict more easily what she wants*. (P16)

In particular, the inclusion of wraparound workshops such as baby massage, weaning and play and development, gave mothers the impetus to anticipate their infant’s needs and to encourage their development:

*We had a lot of direction around upcoming milestones and what you would need to be focusing on to make sure that your child is going to achieve them… about brain development and I got a huge amount out of it and so did [baby]* (P20)

Having the babies present during the programme was viewed as hugely beneficial both in terms of seeing how other infants were in the group were developing at different rates and also for allowing mothers to put apply new skills with their own babies:

*Week to week when you’re physically seeing the babies there, so I got to see other people’s babies and how they were growing and developing and they fed back what they could see with [my baby]. Like that she was changing, growing, getting bigger, had learned how to do something …* (P11)

All mothers praised the facilitators for their constant support and encouragement. They commended the approachability of facilitators in providing a safe and welcoming environment where everyone’s opinion was valued:

*The public health nurse was so nice, lovely, really welcoming … really happy, delighted to see us and telling us we were brilliant for getting out of the house with baby*. (P15)

*Because the nurses are there and you knew they were Public Health Nurses, they look after babies, you’re confident that the babies are safe*. (P21)

***(c) Increased awareness of infant development***

Interviews with the mothers suggested that, as a result of their engagement with the programme, they became increasingly aware of the importance of early interaction, and communication, in supporting infant skill development. Initially, many mothers believed that their baby could not understand, or benefit, from interaction at such a young age. As a consequence of programme participation, many felt encouraged to communicate more with their baby in order to promote their language and cognitive skills:

*Like your connection with your child. I don’t think people realise, at such an early stage, how important say the parent-ease and stuff like that, even talking to your baby, how important it is for their development*. (P22)

*… the importance of being there with the child and talking and playing … How you interact and communicate with your child has a big impact on how development is … He is very curious, he’s very interested in things and I’m sure that has been because we’ve been more focused on interacting with him a lot more*. (P2)

All mothers reported enhanced interaction and communication with their infants, specifically in terms of more responsive parenting. This suggests mothers were adopting a more proactive approach to fostering infant development. Practical techniques such as mimicking baby’s babbling, pulling expressions, singing and using face-to-face communication rather than a toy, for example, were widely mentioned and appeared to suggest a greater understanding of the importance of greater interaction and communication with infants:

*I didn’t know what to do when they started babbling. Whether to babble back at them or ignore it … So it was suggested that babble back at them or a mixture of both so I’d do the same back at her and supposedly it gives them confidence that what they’re saying is meaningful you know and that they’re being listened to*. (P9)

*She definitely picked up something from me speaking very kind of descriptively to her even though I didn’t know the benefits of it at the time. And that’s certainly something I’d never have considered doing with a new baby*. (P21)

*When they told us stuff like your baby loves watching your face, rather than any toy and playtime for them could literally be sitting on your knee and talking to them and pulling funny faces … that’s not something I’d heard anywhere before. That give me a lot more confidence … instead of thinking oh I have to put her in a bouncer or on a playmat, that it’s fine to just sit her on my knee and that she actually she might get more enjoyment out of that than anything else*. (P16)

**3. Programme challenges**

Overall, there were few negative reports from mothers on the programme. Primarily, criticisms were levelled at the outdated nature of the vignettes and the corresponding lack of an Irish perspective. A couple of parents suggested that the emotional aspect of returning to work could have been incorporated into the Return to Work workshop which primarily focused on the practicalities and the quality and standards that apply to early childhood care. Two mothers felt the programme was somewhat basic in terms of programme content, although one mother acknowledged *‘I suppose it has to cater for everybody’s level of knowledge’ (P21)*. One parent commented on the lack of information in advance of the programme and suggested a need for greater information on programme content and structure which, in turn, may have raised awareness of programme topics and promoted engagement:

*I think there needs to be a bit of a restructure in how they deliver. I know I got a printed leaflet … I wasn’t sure what exactly was going to happen … how am I going to do this class with a baby? Is the baby going to have to stay in a buggy?* (P1)

Other adverse comments related to the Toddler programme; for example, toddlers could not attend the programme and costs associated with childcare was prohibitive for some. Furthermore, many mothers had returned to work with the result that programme uptake was low. Another parent felt the Toddler programme should be delivered earlier, to allow more time for parents to be better prepared for their infants’ sleeping or language developments.

## Facilitators’ experiences

The analysis of the data provided by the facilitators identified a number of important themes and sub-themes (Table 3).

**Table 3.** Main themes and sub-themes related to facilitator participants

**Relationship building and engagement**
**Perceived benefits for participants**
Supportive environment and parental empowerment
Promoting positive parent–infant relationships
**Perceived benefits for facilitators**
Partnership approach
Enriched work practices
**Programme challenges**

**1. Relationship building and engagement**

The facilitators highlighted the importance of identifying parents who may be in need of, or could benefit from, the programme. Typically, recruitment was undertaken by PHNs at the 6-week developmental check-up, or by staff in local family resource centres. Previously, a number of mothers (*n* = 7) had indicated attending the programme on recommendation by their PHN. One PHN suggested that nurses were well-positioned to recruit participants, given their role in providing postnatal support:

*… the Public Health Nurses are in the best position because they are seeing the mammies. So a GP mightn’t see the mammy at all. Some GPs don’t even do the checks on the babies, it’s the practice nurse*. (PHN Focus Group)

Furthermore, having PHNs co-facilitate the programme was perceived to contribute to greater engagement with the programme, as one non-PHN facilitator remarked:

*But with regards to the facilitators and the public health nurses and that now it works very well and it’s great because a lot of people they just look up to the baby nurse, they know everything. So it’s great, it’s a great mix*. (S3)

The majority of facilitators reported that if they had a good relationship with the mother, she was more inclined to participate and to remain with the programme. Importantly, facilitators observed that parents from higher socioeconomic groups were equally as vulnerable as those from more disadvantaged backgrounds, due to a lack of family and/or social support:

*… they are educated women and they need the programme just as much as a little 16-year-old in a house with their mammy, probably more so because they have no support. The professional women sitting out there is probably miles from their family, miles from friends, maybe none of her friends have babies yet, she has read the book, she is going to do it right, she just doesn’t get it until you sit down on the floor and chat to your baby*. (S5)

In most cases, all participants would have met their facilitator prior to the programme, either in their homes or at coffee mornings arranged in the local community centre. Facilitators felt this initial meeting was crucial to encouraging engagement with the programme, especially amongst more vulnerable mothers:

*I think it’s very important to start a little bit of relationship building before the programme starts. The [local family resource centre] would always be an advocate of meeting parents before a programme starts, having one or two phone calls … it’s always a way of getting just to see that face to face rather than just meeting them on the first morning*. (S3)

**2. Perceived benefits for participants**

***(a) Supportive environment and parental empowerment***

Similar to the parent reports, all facilitators frequently referred to the social benefits of the programme for new mothers, specifically in terms of group interaction and peer support. Likewise, they also reported the effectiveness of social media in facilitating mothers’ friendships during the programme:

*Thank God for social media, it is just fantastic … they have all set up either a WhatsApp group or some sort of a Facebook page and they regularly communicate*. (S5)

There was also a marked emphasis on shared learning and collaboration throughout the programme. Facilitators were keen to encourage discussions and problem-solving among parents in order that they reflect on their own parenting styles and resolve any parenting difficulties. This approach was seen as crucial to developing a sense of empowerment amongst mothers:

*We are all on a learning programme, we are all there to learn from each other and we don’t want to create that environment for them that they might feel threatened in any way*. (PHN Focus Group)

*… I think it needs to be really clear that I am not here to teach you anything, I am here to facilitate it. You are the experts, we are here to guide the situations and introduce topics and that is our role*. (S8)

***(b) Promoting positive parent–infant relationships***

One of the main themes to emerge from the facilitator findings related to their reports of increased bonding and communication between mothers and infants. While practicalities such as feeding and sleeping techniques were covered within the programme, facilitators were keen to highlight the crucial role that parents play in stimulating their baby’s overall psychosocial development. As one stakeholder noted:

*… if parents only realised how much they can shape and influence what these little people grow up and become and take ownership for their part in it. Instead of looking externally to the schools or whatever it may be and if they made the change … then I think that would be huge*. (S7)

Particular care was taken by facilitators throughout the programme to nurture the relationship between mothers and their babies, and to explicitly address the mother–infant relationship with a view to increasing maternal sensitivity towards babies’ cues and developmental needs:

*A lot of it is new, the singing, the nursery rhymes, the stimulation, the brain development, all of that, they come home from hospital with the first baby and they are thinking feeding it, changing its nappy and getting it to sleep, they are really the priorities. So it is just looking at it a little differently …* (S4)

Facilitators also highlighted the benefits of having the infants attend with mothers in facilitating a more ‘hands-on’ approach to improving their baby’s learning. Various techniques were recommended by facilitators to promote infant development; these included sitting on the floor at baby’s eye level, increased eye contact, loving touch and more descriptive communication (i.e., talking about what your infant is doing in greater detail). As the programme progressed, the facilitators observed greater, and more regular, interaction between mother and infant:

*And the way they just change their attitudes. Instead of the baby on its own they are talking, by the fourth, fifth, sixth session, they are on the floor, automatically they sit down on the floor and talk and they will still be talking to you because they are at their baby’s level …* (S5)

**3. Perceived benefits for facilitators**

***(a) Partnership approach***

Several nurses reported that, as a direct result of facilitating the programme, they had adapted their clinical practice to become less instructive and more focused on actively engaging the mother in dialogue around caring for their infant:

*It’s hugely enriched my working with my parents, even the ones who are not doing the groups. I would bring the same sort of ideas around managing different things and working out different things from [the programme]*. (S2)

*I am much less prescriptive, if someone asks a direct question about weaning and they are in a clinic situation, that’s a yes or no, but even with weaning them I would say, ‘well what do you think he might like to eat or why do you think he made that noise?’ As opposed to saying, ‘he is not choking’. It definitely makes you think about how you work with mothers*. (S5)

This collaborative, or partnership, approach to learning required PHNs to shift from the more traditional role of imparting knowledge to the service user, to that of facilitating decision-making and problem-solving in a more inclusive manner:

*As a Public Health Nurse, we go out and if a person is having an issue … we will be saying ‘this is what you do …’ but … through working with the groups, it’s been good … the collaborative model that the parents help each other and that we come in a little bit with our PHN hats on but at the same time letting the people work it out for themselves … it’s the biggest, positive thing for the parent because they end up feeling ‘wow, I can do this’*. (S2)

***(b) Enriched work practices***

Following programme training, facilitators spoke of a greater awareness and knowledge amongst mothers of the psychological well-being of their infants. Moreover, some PHN facilitators observed stronger mother–infant bonds both in group and healthcare settings – such as attending developmental examinations in clinics. Some PHNs felt that mothers who attended the programme were identifiable when they presented for later developmental check-ups. These mothers were seen to be more aware of the value of regular interaction and communication for infant development and consequently, clinic visits were perceived as shorter, more focused and more efficient. Several stakeholders observed greater incidences of parent–infant contact, and more positive, expressive communication by mothers towards their infants:

*… you can see that the mums are far more engaged with them … When they are undressing them and they are doing things… So the way that they are communicating with their baby is very, very different and it’s a lot more descriptive in nature … my colleagues have noticed that as well*. (PHN Focus Group)

One PHN facilitator suggested that infant psychosocial development be taught as part of the public health nursing programme – both to complement, and to augment, the more clinical aspects of nursing:

*… I think it is an awful shame that it is not part of the public health nursing programme, that this isn’t something that every public health nurse is exposed to and it takes away the clinical side of nursing. There is much more nurturing and I think it is so important when you deal with babies, so important*. (S5)

**4. Programme challenges**

Facilitators noted that the vignettes were the least popular component of the IYBP programme and recommended that they be modernised and updated; others remarked on participants’ dislike of role-plays; *‘it is always the thing that scores low on the evaluation’* (S8).

The recruitment and engagement of more vulnerable and higher risk parents as well as younger mothers (under 23 years old) was also mentioned as a significant barrier to recruitment:

*It can be hard to sell to certain profiles. As I say … certain vulnerable clients, to certain non-nationals, non-Irish people… You know, it can be hard to get a mixed profile really*. (S2)

In addition, the challenges associated with modifying parenting behaviour and attitudes was widely acknowledged by facilitators, both in instances of ‘hard to reach’ mothers, but also for ‘middle-class’ mothers with preconceived, or idealised parenting beliefs:

*Definitely where a person is coming from, what their experience as a child has been effects how they parent …. our hard to reach mums would be particularly like that … They are the hardest to change … I think there’s also the opposite end … a whole new sort of cohort of yummy mummy type of behaviour where they will have researched everything on the internet and they will be following different programmes for parenting … It can be hard to break that… To change that thinking*. (S2)

Some facilitators spoke of the length of the IYBP as a barrier for some participants. It was perceived as a huge time commitment for new mothers, but at the same time, facilitators were keen to highlight that early engagement and a good relationship with the participant often facilitated recruitment and ongoing participation. Facilitators also referred to the lack of follow on to the Toddler programme, but this was seen as a consequence of many participants returning to work. Likewise, childminding was mentioned as a barrier to attending some of the workshops such as the weaning, first aid and toddler programmes. Two PHN facilitators spoke of the challenge of adopting a new teaching approach and set of skills for group delivery. Both PHNs had no previous experience of facilitating a group-based programme and initially found the training overwhelming:

*I’d never been involved in group work so actually coming out of my safe place to stand up or sit down even and talk to a whole group the whole thing was quite a big learning curve for me … I was working in a programme with psychologists in the beginning … They would be very used to it, … so I would have learnt a lot from them but would have been very intimidated at the same time trying to work… Very different from our role as a nurse. Very, very different*. (S2)

*But we were on a learning curve first time around whereas this time we are a little bit more relaxed. We are a little bit more comfortable in our skin doing it. We are not concentrating on pieces of paper in front of us and it’s free flowing a little bit more and you are trying to get them to actually look at themselves as being the parent*. (PHN Focus Group)

Interestingly while these PHNs respected the role of trainers in developing their group facilitation skills, this was seen by some as a mutually beneficial exercise, whereby the expertise of all involved contributed to the learning process. For instance, the knowledge and skillset of the PHNs were highlighted and appreciated by one experienced, community-based, facilitator:

*They [the PHNs] nearly look up to us, they really do sometimes and I’d be looking up to them because I’m going, sure they [have the] world’s knowledge around babies and midwifery and … when it comes back down to it, it’s about how you’re dealing with the people and how you are facilitating the information, you know it’s not about telling them what to do or telling them how to do it*. (S3)

## Discussion

The overarching aim of this study was to assess whether an early parenting intervention delivered by community-based health professionals on a universal basis within disadvantaged areas – and comprising locally-based programmes and workshops to promote learning and development – was seen as beneficial to new mothers. A secondary aim was to explore facilitators’ perceptions of benefits for participants and identify learning and/or barriers to programme acceptability. Firstly, the study findings provide important insights into the emotional state of mothers, and the profound changes they experience as they restructure their lives and adjust to motherhood. While this was not the primary focus of the study, it was important, nonetheless, to ask mothers about their experiences in the early months after the birth of their baby. Perhaps unsurprisingly, mothers expressed feelings of inadequacy, powerlessness, exhaustion and anxiety in the immediate months post-birth. These findings are consistent with existing research on early parenthood (Deave, Johnson & Ingram, 2008; Sanders, Lehmann & Gardner, 2014) and demonstrate how, in spite of the availability of information and care provided by healthcare professionals, parents are not always prepared or equipped for motherhood and often experience feelings of stress and insecurity. In both the above studies, participants’ engagement with supports was varied, but indicated a need for more timely and relevant interventions to improve the experiences of motherhood. Specifically, the practical aspects in caring for their baby, and the attendant impact of motherhood on their lives, emerged as real concerns. Parental reports from this study suggests that a complex early intervention programme, such as the PIN – delivered in community settings as part of routinely delivered services and in line with infant development – offers important practical and emotional supports to minimise the difficulties associated with transitioning to motherhood.

The findings also highlight the importance of exploring the perspectives of both programme users and providers in understanding why participants engage with a programme, whilst also identifying specific programme components perceived to be of most (or least) use. Mothers described the benefits associated with baby massage, weaning, paediatric first aid and play and development, while programme materials, such as the weekly notes, handouts and homework, were widely considered useful. A number of common themes also emerged across both groups (parent and facilitator). These include: the importance of responsive parenting and empowering the parent; peer learning and collaboration; the importance of social networks in promoting positive mental health; and the ways in which increased interaction and communication can help to promote infant socioemotional development. The findings also suggest that the PIN programme helped to alleviate concerns, anxiety and stress surrounding the transition to the role of parenthood. Social support was found to be the single most important factor for engaging mothers across both groups and was perceived to be essential for increasing mothers’ parental sense of competence and confidence. This is particularly important as new mothers often experience isolation due to a lack of support from extended family, or a social network (Paris & Dubus, 2005), resulting in fewer opportunities to learn about caring for their infant (Hanna *et al*., 2002). Mothers in our study emphasised the value of support from extended family, particularly partners/husbands and the maternal mother. The programme was also considered valuable in addressing postnatal depression and anxiety. Mothers described coping better and feeling more confident as a result of their engagement with the programme.

Peer-led learning and support were also identified as important to strengthening parenting skills and competency. Mothers shared effective strategies for managing their infants’ behaviour with other parents and were encouraged to discuss and practise trialling new ways to manage their baby’s sleep or feeding patterns/routines. Indeed, peer support further allowed mothers to openly discuss any topics or issues of concern and was pivotal in normalising the difficulties associated with motherhood and enhancing mental well-being. The use of social media also reinforced the connections between mothers, enabling them to build relationships, often after programme completion. These findings are consistent with some existing research which shows that parenting programmes can contribute to the psychosocial health and well-being of mothers (Barlow, Coren & Stewart-Brown, 2003). Challenges concerning the outdated and unrealistic nature of the vignettes and the lack of childcare for some programme components (i.e., Toddler programme) have previously been identified as barriers to attendance (Stern *et al*., 2008; Trillingsgaard *et al*., 2014). Adapting programme materials so that they are culturally relevant (Baumann *et al*., 2015), ensuring programmes are accessible, and providing free childcare and transportation, could minimise attrition and encourage greater overall engagement (Axford *et al*., 2012). Despite these difficulties, the PIN programme, as a multicomponent intervention, has the potential to optimise the parental and infant well-being through the use of additional workshops and supports. For example, workshops such as baby massage, weaning, first aid and baby play and development, were found to reduce worry and anxiety around infant care and feeding practices, whilst also building parent–child attachments and parental confidence.

The findings from the facilitator interviews also demonstrated the importance of establishing an informal relationship with mothers prior to programme commencement to maintain engagement and reduce drop-out. Recruitment difficulties were highlighted by some facilitators, particularly for families who were considered more at risk, as well as non-Irish families. Engaging more vulnerable families in programmes is frequently identified as a barrier to participation, and building a positive relationship with families facilitates recruitment and ongoing engagement (Prinz *et al*., 2001; Moran, Ghate & van der Merwe, 2004; Landers *et al*., 2018). In this study, recommendations by community organisations and PHNs to attend the programme, were viewed as having positively influenced recruitment and minimised drop-out. For example, a large number of mothers who participated in the programme did so on a recommendation from their PHN or family resource/local health centre. Thus, the involvement of community healthcare providers/practitioners facilitated the recruitment and engagement of more vulnerable or disadvantaged participants and indeed, parent reports suggest good working relationships with facilitators throughout the programme.

Accordingly, PHN facilitators spoke of adopting a partnership approach in working with parents which required a shift in focus from one of an ‘expert’ imparting advice to mothers, to one of facilitating peer learning and encouraging the sharing of knowledge (Fowler *et al*., 2012). Similar findings were echoed by the mothers who were highly complimentary of the facilitators’ delivery, both in terms of creating a welcoming and non-judgemental environment and for facilitating shared learning amongst the group. This reflects the findings by Barlow and Stewart-Brown (2001) and Kruske *et al*. (2004) who reported that parents found it more helpful if they were supported in their parenting role rather than being told how to parent. Some PHNs, however, found the experience of delivering a group programme intimidating, and acknowledged the often difficult transition from the role of PHN to facilitator. Nevertheless, they were very positive about the peer support they had received and reported feeling more comfortable with delivery as the programme progressed. Overall, our findings underscore the important role both community development workers and PHNs play in identifying mothers in need and supporting them through the early stages of motherhood. Positive engagement can also lead to a better relationship between parent and health professionals which, in turn, increases the likelihood of parents accessing additional community supports thereby promoting further health outcomes for mothers and children.

Following programme participation, mothers spoke of being more sensitive and responsive to their infants’ emotional needs and reported having a more proactive role in their development. Techniques such as singing, playing and reading to their infant were introduced earlier in order to encourage and stimulate their infant’s socioemotional and cognitive development. Some mothers of older infants (> 1 year old) spoke of improvements in their infants’ cognitive and language ability which they attributed to greater communication with their infant. Equally, facilitators remarked on increased parental confidence and self-esteem as well as greater use of positive communication between PIN, thereby suggesting the programme empowered mothers in their role as parents. The facilitators also observed greater mother–child interaction at clinic visits. Evidence by Barlow *et al*. (2010a) suggests that positive parent–infant interaction during the postnatal period, is closely linked to neurological development and later socioemotional functioning; these authors recommend, therefore, that parent–infant interaction be included alongside existing evidence-based partnership models in order to improve child outcomes.

## Implications for practice

The findings highlight the needs of women as they prepare for motherhood and underscore the requirement for earlier supports to address those needs. Furthermore, given the adverse outcomes associated with postpartum depression and poor mother–infant attachment (Murray *et al*., 1996; Field, 2010; Haga *et al*., 2012), they further reinforce the need for earlier identification and more targeted interventions during the postnatal period. Supporting the transition to parenthood by providing effective, and accessible, maternal services will benefit the most disadvantaged and deprived within our communities (WHO, 2014). Health and community care practitioners are well placed, therefore, to identify more vulnerable families by establishing positive relationships and encouraging programme engagement (Prinz *et al*., 2001). Furthermore, interventions that promote and incorporate early child development into existing health services, are recommended to mitigate the effects of multiple disadvantage, including poverty (Engle *et al*., 2007). Mothers reported high levels of programme satisfaction and valued the peer support for promoting their parenting capabilities and well-being. The PHN facilitators reported integrating learning from programme delivery into their own routine practice, and tangible improvements were reported at developmental check-ups; mothers were perceived as more confident and knowledgeable of their infants’ development, making clinic visits quicker and more focused. This has important implications for service efficiency. PHNs describe a more comprehensive assessment at infant developmental appointments that combines more traditional clinical expertise with infant mental health principles, thereby facilitating their workload and capacity to strengthen parenting skills.

## Study strengths and limitations

This study used a purposive sample which may limit the generalisability of the findings. However, sample size included both first-time and second-time mothers of varying ages, who were recruited across two delivery sites. Mothers were interviewed at different stages of their infants’ development in order to capture their experiences of programme delivery during the lifetime of the programme. While feedback from participants was generally very positive, additional interviews with parents who dropped out of the programme may have provided information on the challenges they encountered, or programme components with which they were less satisfied. Similarly, no fathers participated in this study, and while this is reflective of parenting programmes overall (Tiano and McNeil, 2005), little is known about fathers’ influence on brain and child development (Cabrera, Volling & Barr, 2018). Future research in this area would be important in helping us to better understand the father’s role within the wider context of childrearing.

Data were collected prospectively from a large sample of key stakeholders (including parents and programme providers) as the programme was delivered, and is reflective, therefore, of the ‘real time’ experiences of the PIN programme. However, in some instances, the focus groups comprised smaller than anticipated numbers which may have influenced, at least to some extent, the group dynamic. For example, in site 2, programme implementation was in the early stages of delivery and only a small number of PHNs had been trained in the programme. In another case, there were practical challenges in bringing people together due to competing workloads and time constraints.

## Conclusion

The findings reported here provide important insights into the reasons why parents engage with an early intervention and prevention programme in the first weeks after birth and what they perceive to be of value in terms of early parenting supports. Understanding when and why parents engage with programmes of this nature is important and can help to inform the development of effective engagement and delivery strategies, particularly for more vulnerable, or high risk, parents. The findings suggest that programmes such as the PIN, can provide important practical and emotional support to foster parental confidence and promote parent–child attachment. Parents are often ill-prepared for parenting (e.g., Sanders, Lehmann & Gardner, 2014) but the mothers in our study, reported high levels of satisfaction with the PIN programme and its delivery, as well as perceived benefits such as reduced parental stress and isolation and improved parental capacity. Improvements in parent–infant responsiveness and the parent–infant relationship were widely reported by parents and facilitators alike. Our findings further underline the need for early supports in promoting mothers’ confidence and satisfaction with parenting (Börjesson, Paperin & Lindell, 2004). PHN facilitators described how the programme enhanced their own professional expertise and practice and contributed positively to their understanding of infant psychosocial well-being. The findings also point to the potential value of integrating group-based parenting programmes into primary health care settings with additional benefits for improved routine clinical practice and nurse professional development, thereby contributing to sustainability, whilst also empowering parents to address the needs of their infants and their own personal well-being. This reflects findings by Hurt and colleagues (2018) who recommend that interventions (up to 24 months) delivered within existing healthcare settings have the potential to improve interaction with parents and healthcare professionals, improve access to services and ultimately support parents to promote child development outcomes (Hurt *et al*., 2018). Nonetheless, the shift in practice from PHN to facilitator role was somewhat daunting for some PHNs and underlines the challenges some healthcare practitioners may experience in adapting to a new model of care requiring nurse–client partnership and collaboration (Gallant, Beaulieu & Carnevale, 2002; Fowler *et al*., 2012). This study forms part of an ongoing large-scale research programme and our further work will explore the effectiveness of the PIN programme in improving parent sense of competence and well-being, parenting skills and child development when compared to services as usual, whilst we will also explore implementation fidelity and the cost-effectiveness of the programme.
